# The complete chloroplast genome sequence of *Bambusa beecheyana* var*.pubescens* (Bambusodae)

**DOI:** 10.1080/23802359.2020.1823275

**Published:** 2020-10-05

**Authors:** Liguang Chen, Yangyang Zhang, Lili Fan, Lingyan Chen, Tianyou He, Yushan Zheng

**Affiliations:** a College of Forestry, Fujian Agriculture and Forestry University, Fuzhou, P. R. China; b College of Arts & College of Landscape Architecture, Fujian Agriculture and Forestry University, Fuzhou, Fujian, P. R. China

**Keywords:** *Bambusa beecheyana* var. *pubescens*, Plastid genome, Phylogeny, Bambusodae

## Abstract

*Bambusa beecheyana* var*. pubescens* is mainly distributed in South China to Southwest China, growing on hillsides or river banks. In the current study, we sequenced the complete chloroplast genome of *B.beecheyana* var. *pubescens* and reported for the first time. The genome is 139,402 bp in total length, include a large single-copy (LSC) region of 82,936 bp, small single-copy (SSC) region of 12,868 bp, a pair of invert repeats (IR) regions of 21,799 bp. Plastid genome contains 132 genes, 85 protein-coding genes, 39 tRNA genes, and 8 rRNA genes. Phylogenetic analysis based on 25 chloroplast genomes indicates that *B. beecheyana* var*. pubescens* is closely related to *Bambusa oldhamii*, *Bambusa ventricosa* and *Bambusa ventricosa multiplex* in Bambusodae.

*Bambusa beecheyana* var*. pubescens* (http://www.theplantlist.org/) is one of the taller bamboo species with the developed and simple planting method to establish. It is an outstanding bamboo species for landscaping and soil and water conservation. The chloroplasts (cp) genome has a maternal inheritance and conserved structure, that has been used to examine the developmental and phylogenetic relationships of plants. (Wang et al. 2018).Therefore, we reported the complete cp genomeof *B. beecheyana* var*. pubescens* based on Illumina pair-end sequencing data. Fresh leaves tissues of *B. beecheyana* var*. pubescens* were collected from Fujian province, China (Fujian Agriculture and Forestry University, Bamboo Garden, Fuzhou:119°14′16″E, 26°5′7″N), and dried into silica gel instantaneously. The specimens were preserved in the Herbarium of College of Forestry, Fujian Agriculture and Forestry University (specimen code HTY021). DNA was extracted from fresh leaves tissues, while its quantification was verified using Agarose gel electrophoresis and concentration Nanodrop, with 500 bp randomly interrupted sequence by the Covaris ultrasonic breaker for library construction. Approximately, 2.0 GB of raw data were generated with 150 bp paired-end read lengths. The Illumina High-throughput sequencing platform (HiSeq2500) data were filtered by the script in the NOVOPlasty (Dierckxsens et al. 2017).The complete plastid genome of *Salix rehderiana* (GeneBank accession: MG262367) as reference and plastid genome of *B. beecheyana* var*. pubescens* were assembled by GetOrganelle pipe-line (https://github.com/Kinggerm/GetOrganelle), it can get the plastid-like reads, and the reads were viewed and edited by Bandage (Wick et al. 2015). The cp genome annotation was assembled based on the comparison by Geneious v 11.1.5 (Biomatters Ltd, Auckland, New Zealand) (Kearse et al. 2012). The annotation results were drawn with the online tool OGDRAW (http://ogdraw.mpimp-golm.mpg.de/) (Lohse et al. 2013).

The complete cp genome of *B. beecheyana* var. *pubescens* (GenBank accession: MT843581) was 1,39,402 bp in length, with a large single-copy (LSC) region of 82,936 bp, a small single-copy (SSC) region of 12,868 bp, and a pair of inverted repeats (IR) regions of 21,799 bp. The complete cp genome consisted of 132 genes, having 85 protein-coding genes, 39 tRNA genes, and 8 rRNA genes. The complete cp genome GC content was 38.92%. In order to reveal the phylogenetic position of *B. beecheyana* var*. pubescens* with other members of Bambusodae, we performed a phylogenetic analysis based on 23 complete cp genomes of *Bambusodae*, and two taxa (*Arundinaria gigantea、Arundinaria fargesii*) as outgroups. All of them were downloaded from NCBI GenBank. The sequences were aligned by MAFFT v7.307 (Katoh and Standley 2013), and the phylogenetic tree was constructed by RAxML (Stamatakis 2014). The phylogenetic tree revealed that *B. beecheyana* var*. pubescens* was most closely related to *B. oldhamii*, *B. ventricosa* and *B.multiplex* with strong support (Figure 1).

**Figure 1.**
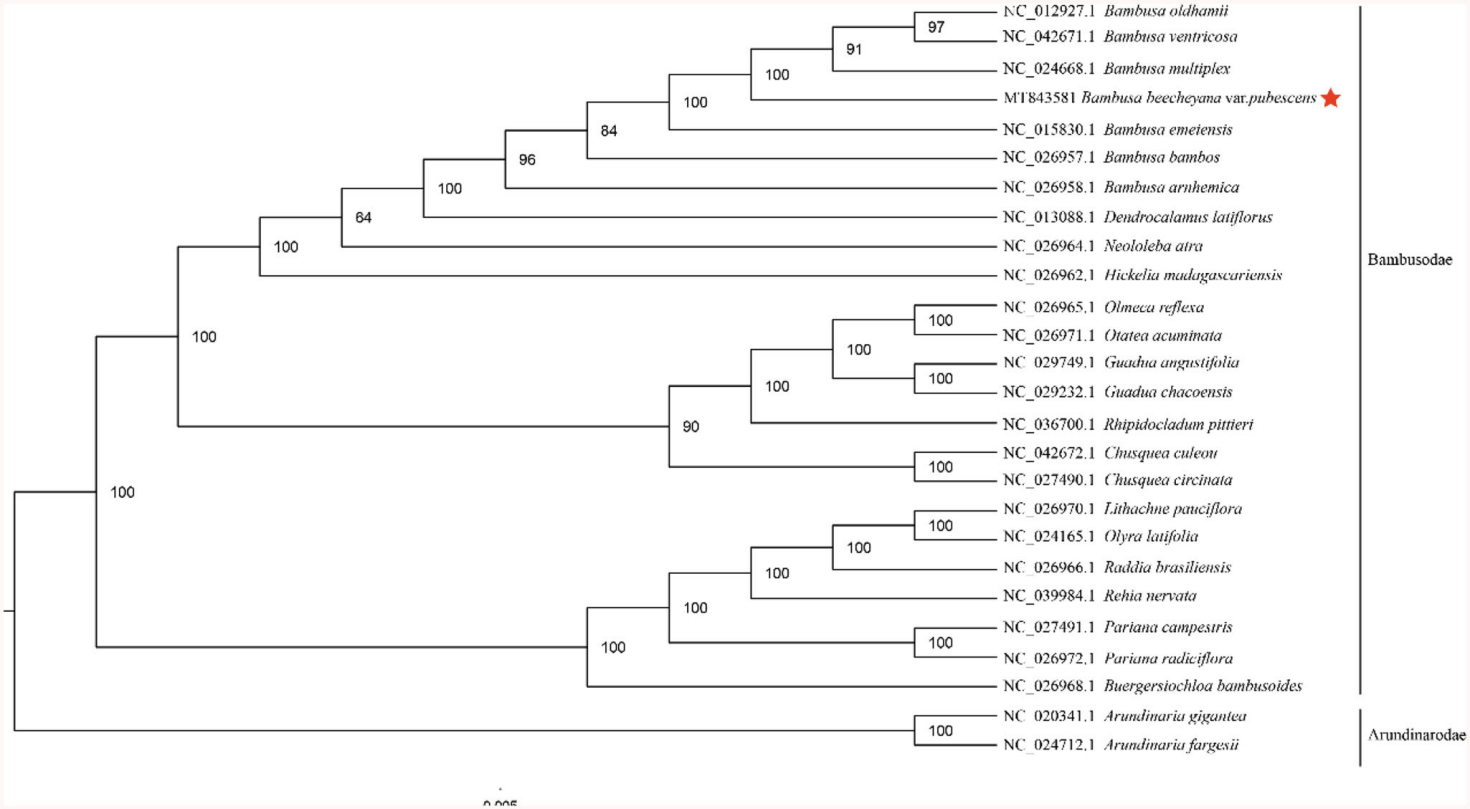
Phylogenetic analysis of 23 species of Bambusodae and two taxa (*Arundinaria gigantea*, *Arundinaria fargesii*) as outgroup based on plastid genome sequences by RAxML, bootstrap support value near the branch.

## Data Availability

The data that support the findings of this study are openly available at https://www.ncbi.nlm.nih.gov/ GeneBank with following accession number; MT843581 (BankIt 2370800 HTY021).
